# Methylation-related metabolic effects of D4 dopamine receptor expression and activation

**DOI:** 10.1038/s41398-019-0630-3

**Published:** 2019-11-12

**Authors:** Nathaniel W. Hodgson, Mostafa I. Waly, Malav S. Trivedi, Verna-Ann Power-Charnitsky, Richard C. Deth

**Affiliations:** 1 Department of Neurology and the F.M. Kirby Neurobiology Center, Boston Children’s Hospital, Boston, MA 02115 USA; 2 Department of Food Science and Nutrition, College of Agricultural and Marine Sciences, Sultan Qaboos University, Muscat, 123 Oman; 3 Department of Pharmaceutical Sciences, Nova Southeastern University, Fort Lauderdale, FL 33328 USA; 4 Department of STEM, Regis College, Weston, MA 02493 USA

**Keywords:** Molecular neuroscience, Pathogenesis

## Abstract

D4 dopamine receptor (D4R) activation uniquely promotes methylation of plasma membrane phospholipids, utilizing folate-derived methyl groups provided by methionine synthase (MS). We evaluated the impact of D4R expression on folate-dependent phospholipid methylation (PLM) and MS activity, as well as cellular redox and methylation status, in transfected CHO cells expressing human D4R variants containing 2, 4, or 7 exon III repeats (D4.2R, D4.4R, D4.7R). Dopamine had no effect in non-transfected CHO cells, but increased PLM to a similar extent for both D4.2R- and D4.4R-expressing cells, while the maximal increase was for D4.7R was significantly lower. D4R expression in CHO cells decreased basal MS activity for all receptor subtypes and conferred dopamine-sensitive MS activity, which was greater with a higher number of repeats. Consistent with decreased MS activity, D4R expression decreased basal levels of methylation cycle intermediates methionine, S-adenosylmethionine (SAM), and S-adenosylhomocysteine (SAH), as well as cysteine and glutathione (GSH). Conversely, dopamine stimulation increased GSH, SAM, and the SAM/SAH ratio, which was associated with a more than 2-fold increase in global DNA methylation. Our findings illustrate a profound influence of D4R expression and activation on MS activity, coupled with the ability of dopamine to modulate cellular redox and methylation status. These previously unrecognized signaling activities of the D4R provide a unique link between neurotransmission and metabolism.

## Introduction

**Fig. 1:**
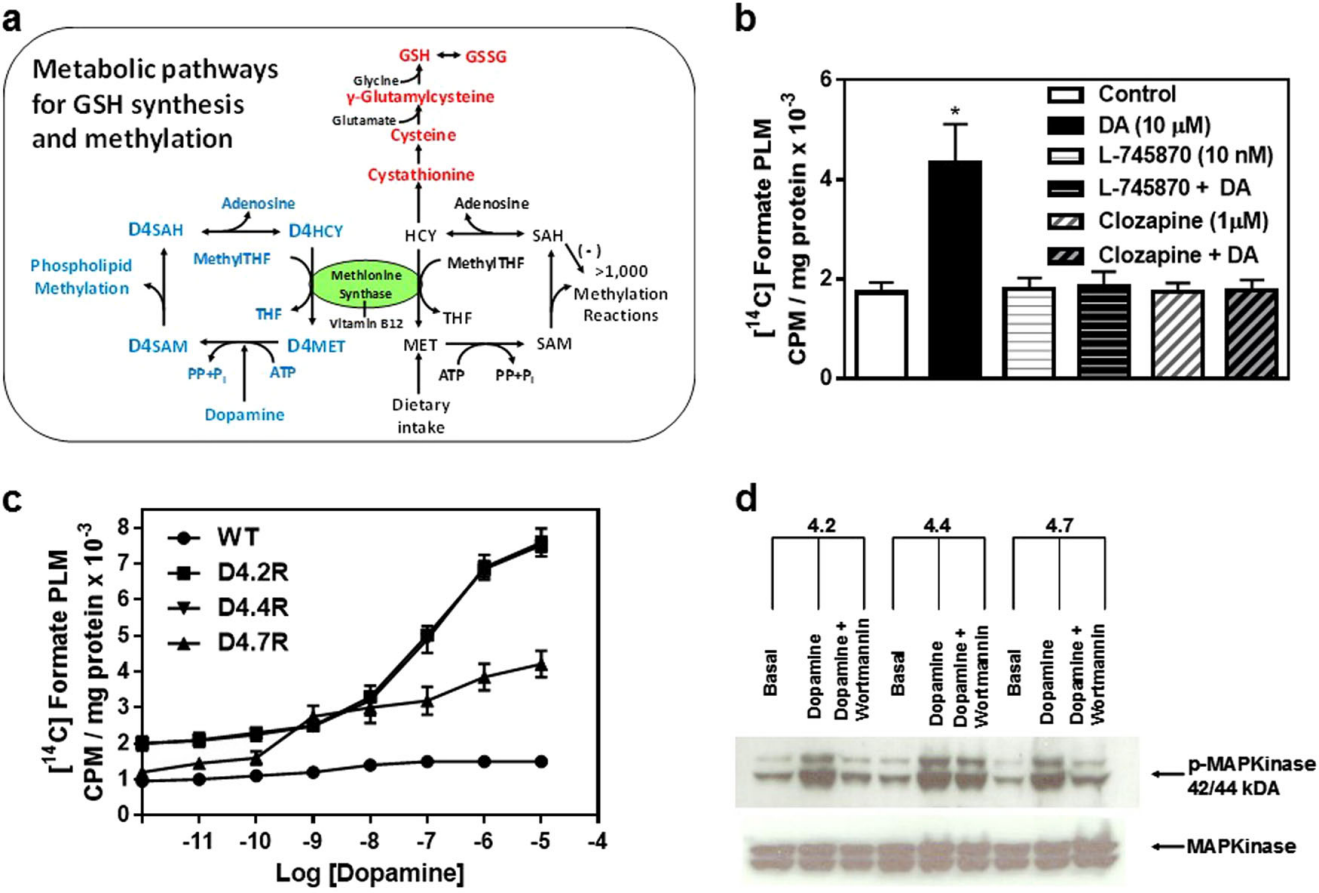
Dopamine-stimulated PLM in D4R-expressing CHO cells. **a** The dopamine-stimulated D4R-mediated PLM cycle (lower left) functions in parallel to the canonical methionine cycle (lower right) and is completely dependent on MS activity and 5-methyltetrahydrofolate (methylTHF). Transsulfuration of homocysteine to cystathionine and cysteine supports GSH synthesis. **b** D4.4R-expressing CHO cells were treated with dopamine (DA) for 30 min with or without pretreatment with the highly D4R-selective antagonist L-745870 or the moderately selective D4R antagonist clozapine. Data are mean ± SEM of two experiments (*n* = 6). *p < 0.05 vs. Control. **c** Non-transfected CHO cells (WT) and CHO cells expressing D4.2R, D4.4R, and D4.7R were treated with graded concentrations of dopamine. Data are average (±SEM) of two experiments (*n* = 6). (**c**) (*p* < 0.05). **d** CHO cells expressing D4.2R, D4.4R, or D4.7R were treated with dopamine (10 µM; 30 min) or dopamine + the PI3 kinase inhibitor wortmannin (100 nM; 60 min). A Western blot was probed with anti-phospho-MAP kinase (upper panel) or anti-MAP kinase antibodies (lower panel)

Previous studies demonstrated the unique ability of D4 dopamine receptors (D4Rs) to carry out phospholipid methylation (PLM) in response to dopamine stimulation.^1–4^ PLM activity involves conformation-dependent methyl donation by a methionine side chain in the cytoplasmic extension of transmembrane helix #6 (M313 in the D4.4R), in a cyclic process analogous to the well-described methionine-based methylation cycle (Fig. 1a). The D4R-mediated PLM cycle depends on replenishing methyl groups from 5-methyltetrahydrofolate (methylTHF) provided by methionine synthase (MS), and also requires methionine adenosyltransferase (MAT) and S-adenosylhomocysteine hydrolase (SAHH).^1^ As illustrated in Fig. 1a, MS transfers a methyl group from methylTHF to restore the D4R methionine residue (D4MET), in addition to its canonical role in converting free homocysteine (HCY) to methionine. These two competing reactions imply that D4R-mediated dopamine-stimulated PLM activity could influence canonical S-adenosylmethionine (SAM)-dependent methionine cycle activity.

Cobalamin (vitamin B_12_), the cofactor for MS, is easily oxidized,^5^ making both HCY methylation and D4R-mediated PLM highly sensitive to oxidative stress. When MS activity decreases during oxidative stress, a greater proportion of HCY is converted to cysteine via transsulfuration, augmenting synthesis of the antioxidant glutathione (GSH) (Fig. 1a). Decreased MS activity also leads to depletion of SAM, the methyl donor for well over 200 different methylation reactions, including D4R-independent PLM and DNA methylation. Furthermore, S-adenosylhomocysteine (SAH), a product of methylation reactions, retains high affinity for methyltransferases and inhibits methylation reactions.^6^ Hence, D4R-mediated PLM activity could potentially affect the SAM/SAH ratio and cellular methylation status, exerting a broad influence throughout cellular metabolism.

The human dopamine D4 receptor gene (*DRD4*) displays a remarkable number of polymorphisms, some of which affect receptor protein structure.^7–15^ Among these, a 48-bp variable-number imperfect tandem repeat (VNTR) in exon III has generated particular interest, and the human *DRD4* contains from 2 to 11 repeats, displaying a distinctive ethnic and geographic distribution pattern.^12^ The four-repeat variant (D4.4R) is predominant in most populations, with a world-wide allelic frequency of 64%, followed by seven-repeat (25%) and two-repeat variants (5%). The VNTR encodes 16-amino acid proline-rich segments in the third cytoplasmic loop of D4Rs that participate in SH3 domain-dependent linkage of signaling proteins to the receptor.^14^ A higher number of repeats implies that additional proteins can associate with the D4.7R, with the potential for more diverse signaling, although the additional protein interactions can restrict D4R access to phospholipids. The D4R is bound to postsynaptic scaffolding protein-95 (PSD-95) via SH3 domain-based binding, where it modulates *N*-methyl-d-aspartate (NMDA) receptor function in a VNTR-dependent manner,^16^ raising the possibility that the D4R may modulate synaptic function via the influence of dopamine-stimulated PLM on proteins sharing its membrane environment.

Modest differences in G protein/effector coupling efficiency^15,17–19^ and receptor heterodimerization^20,21^ have been described among D4R VNTR variants, as well as greater inhibition of NMDA receptor function in the prefrontal cortex by the D4.7R.^16^ However, the functional importance of D4R repeat number remains unclear and the influence of D4R VNTR on D4R-mediated PLM has not been previously investigated.

In the current study, we examined the impact of D4R expression and dopamine exposure on D4R-mediated PLM and MS activity, as well as redox and methylation status, using stably transfected CHO cells expressing D4.2R, D4.4R, or D4.7R. Our findings reveal that the D4.7R has weaker maximal PLM activity compared to D4.2R and D4.4R. Furthermore, D4R expression suppresses MS activity and confers a previously unappreciated ability of dopamine to exert metabolic and epigenetic regulation, significantly expanding the D4R signaling repertoire.

## Materials and methods

### Materials

Wild-type (WT) CHO cells and transfected CHO cells stably expressing D4 receptors (250–350 fmol/mg protein) were kindly provided by Dr. Oliver Civelli and their ligand-binding properties have been previously described.^15^ All studies were conducted with cells from the first 15 passages. Using designations from Lichter et al.^8^, repeat sequences in the receptors were: D4.2R:αζ; D4.4R: αβδζ; D4.7R: αβηεβεζ; and each contained the duplicated 12 bp sequence in exon I. Dopamine, L-745870, and other chemicals were purchased from Sigma-Aldrich (St. Louis, MO). Antibodies against phosphorylated and non-phosphorylated forms of mitogen-activate protein (MAP) kinase (catalog # 4370 and 9102) were purchased from Cell Signaling Technologies Inc. (Beverly, MA).

### Thiol and thioester quantification

Cells were treated as indicated for individual experiments, after which media were aspirated and cells were washed 2× with 1 mL of ice-cold Hanks’ balanced salt solution (HBSS). HBSS was aspirated from the cells and 0.3 mL ice-cold dH_2_O was added to each well. The cell suspension was sonicated for 15 s on ice, and 100 μL of sonicate was removed and used to determine protein content. Two hundred microliters of the remaining sonicate was added to 50 μL of 0.4 N perchloric acid. Sonicates were blown with nitrogen and spun at 13,000 RPM for 60 min to remove protein and cellular debris. One hundred microliters of sample was added to a conical micro autosampler vial, blown with nitrogen, capped, and kept at 4 °C in the autosampler cooling tray (ESA model 542). Ten microliters of sample was injected into an ESA CoulArray high-performance liquid chromatographic system with a BDD analytical cell (model 5040) electrochemical detector at an operating potential of 1500 mV, equipped with an Agilent Eclipse XDB-C8 (3 × 150 mm^2^, 3.5 μm) reverse-phase C8 column. A dual mobile phase gradient elution was used, consisting of mobile phase (A) containing sodium phosphate 25 mM and 1-octanesulfonic acid 2.1 mM, adjusted to pH 2.65 with phosphoric acid, with the second mobile phase (B) containing 50% acetonitrile. The system was run at a flow rate of 0.6 mL/min at ambient temperature with the following gradients: 0–9 min, 0% B; 9–38 min, 30% B. Post run, the system was cleaned with 100% B for 4 min, the cell was cleaned for 3 min at an operating potential of 1900 mV, and the system was equilibrated at 0% B for 8 min prior to the next run. Peak area analysis was provided by CoulArray 3.06 software (ESA, Chelmsford, MA), based on standard curves generated for each compound and sample metabolite values were normalized to protein content.

### MS assay

After drug treatment, cells were scraped, pelleted, and frozen at −80 °C prior to assay. Approximately 10^8^ cells were resuspended in 1 mL of 100 mM phosphate buffer, pH 7.4, containing 0.25 M sucrose. Cell membranes were disrupted by sonication on ice and the homogenate centrifuged at 4 °C. Enzyme assays were performed under anaerobic conditions, as previously described^18^. The reaction mixture contained 100 mM potassium phosphate, pH 7.2, 500 μM HCY, 152 μM SAM, 2 mM titanium citrate, 250 μM (6*R*,*S*)-5-[^14^C]methyltetrahydrofolate, and enzyme in a final volume of 1 mL. Different cobalamins were added at a final concentration of 10 μM. The reaction was initiated by the addition of methylfolate, incubated for 60 min at 37 °C, and terminated by heating at 98 °C for 2 min. Radiolabeled methionine was separated on a Dowex 1-X8 column, which was eluted with 2 mL of water. Control assays, in which sample enzyme was omitted, served as blanks.

### DNA methylation analysis

DNA was isolated using the DNeasy^®^ Blood & Tissue Kit (Qiagen) according to the provided protocol. Isolated DNA was quantified using an ND-1000 NanoDrop spectrophotometer. Isolated DNA was adjusted to a concentration of 50 ng/mL and 100 ng of DNA was used for each assay. Global DNA methylation was assayed using the MethylFlash™ Methylated DNA Quantification Kit (Epigentek).

### PLM measurement

CHO cells were grown in six-well plates in α-MEM supplemented with 10% fetal bovine serum and 1% penicillin/streptomycin/fungizone. After a wash with HBSS, cells were incubated at 37 °C for 30 min or the desired time in 600 μL of Hank’s solution containing 1 μCi/mL [^14^C]formate or [^3^H-*methyl*]methionine, with or without drugs as desired. The reaction was terminated by an initial wash with ice-cold unlabeled HBSS, followed by 500 μL ice-cold 10% TCA. After scraping, cells were transferred to an Eppendorf tube, sonicated, and an aliquot was removed for protein assay. Following centrifugation, the pellet was dispersed in 1.5 mL of 2 N HCl/MeOH/CHCl_3_ (1:3:6), vortexed, and allowed to separate. The lower CHCl_3_ layer was washed twice with 400 μL of 0.1 N KCl in 50% MeOH and an aliquot counted for radioactivity after evaporation.

### Western blot

After treatment, 50 µg of cell lysate was subjected to electrophoresis on a 10–20% sodium dodecyl sulfate-polyacrylamide gel electrophoresis gel. Separated proteins were transferred to a nitrocellulose membrane. Blots were rinsed, blocked, and incubated with primary antibody for 2 h at room temperature with shaking. The blot was then washed and incubated with goat anti-rabbit peroxidase for 1 h, washed, incubated with a chemiluminescence reagent for 1 min at room temperature, and then exposed to autoradiograph film for 10 s.

### Statistical analyses

Figures reflect mean values (±SEM) from representative, replicated experiments. Number of samples is specified in figure legends. Prism 6 (GraphPad Software, San Diego, CA) was utilized for curve fitting and other data analyses. Student’s *t* test (two-sided) was used for statistical evaluation of significant differences, with *p* ≤ 0.05 as a threshold value.

## Results

### Dopamine-stimulated PLM

Dopamine-stimulated PLM was measured using a [^14^C]formate-based assay^1^ that radiolabels the methyl group of 5-methylTHF, allowing quantitation of D4R-dependent methylation of phosphatidylethanolamine (PE). Dopamine significantly increased folate-dependent PLM in CHO cells expressing the D4.4R (Fig. 1b). Consistent with D4 receptor involvement, the dopamine-induced increase was blocked by the highly selective D4R antagonist L-745870 and by clozapine, a moderately selective D4 receptor antagonist. In contrast, when D4R-independent PLM was measured using [^3^H-*methyl*]-methionine, no effect of dopamine was observed (data not shown). These results confirm the ability of D4 receptor activation to selectively promote folate-dependent PLM in transfected CHO cells.

We then compared dopamine-stimulated PLM in dose–response studies with D4.2R-, D4.4R-, and D4.7R-expressing CHO cells. Basal PLM activity was ~2-fold higher in cells expressing D4.2R and D4.4R, whereas D4.7R expression did not affect basal PLM (Fig. 1c). Maximal dopamine activation (10 µM) produced a smaller PLM increase in D4.7R vs. D4.2R or D4.4R-expressing cells (*p* < 0.001), although the percent increase above basal (350%) was similar for all three subtypes. EC_50_ (half-maximal effective concentration) values for dopamine stimulation of PLM were 71 ± 12, 67 ± 9, and 0.82 ± 0.14 nM for D4.2R, D4.4R, and D4.7R, indicating that dopamine was significantly more potent (*p* < 0.001), albeit less efficacious, at stimulating PLM via D4.7R. This result is in contrast to inhibition of cAMP formation, where the D4.7R response to dopamine was reported to be less potent but equally efficacious.^16^ Dopamine stimulation of MAP kinase phosphorylation, mediated by G protein signaling, was similar for D4.2R, D4.4R, and D4.7R, further indicating that PLM is a signaling response that differentiates the D4.7R from D4.2R and D4.4 subtypes.

### MS activity

We next compared the ability of dopamine to affect MS activity in control and D4R-transfected CHO cells, measured as the conversion of HCY to methionine. Basal MS activity decreased by ~90% in all three D4R-transfected cell lines, as compared to WT CHO cells (Table 1), consistent with D4R and MS interaction. A 30 min treatment of intact cells with dopamine (10 μM) had no effect on subsequently measured MS activity in WT CHO cells, but significantly increased activity in each of the transfected cell lines, as previously reported for human neuroblastoma cells.^2^ Interestingly, the size of the dopamine-induced increase in MS activity was dependent on the number of D4 receptor repeats, amounting to 88, 141, and 177% above basal activity for D4.2R, D4.4R, and D4.7R, respectively.

**Table 1:** Methionine synthase activity in CHO cell homogenates after treatment of intact cells with dopamine or the D4R antagonist L-745870

Cell type	Treatment	MS activity (pmol/min/mg)	%WT basal
WT CHO	Basal	205 ± 12	100
	Dopamine (10 µM; 30 min)	208 ± 18	101
	L-745870 (100 nM; 60 min)	201 ± 7	98
	Dopamine + L-745870	216 ± 15	105
D4.2 CHO	Basal	20.5 ± 1.8^a^	10
	Dopamine	38.5 ± 3.3^b^	19
	L-745870	6.6 ± 0.7^b^	3
	Dopamine + L-745870	6.9 ± 0.6^b^	3
D4.4 CHO	Basal	21.2 ± 0.5^a^	10
	Dopamine	48.7 ± 4.6^b^	24
	L-745870	6.5 ± 0.5^b^	3
	Dopamine + L-745870	6.7 ± 0.7^b^	3
D4.7 CHO	Basal	18.8 ± 0.8^a^	9
	Dopamine	56.6 ± 4.3^b,c^	28
	L-745870	6.4 ± 0.3^b^	3
	Dopamine + L-745870	6.5 ± 0.5^b^	3

^a^Significantly different from WT CHO basal (*p* < 0.001)

^b^Significantly different from corresponding basal (*p* < 0.01)

^c^Significantly different from D4.2 + dopamine (*p* < 0.05)

Treatment of intact cells with the selective D4R antagonist L-745870 not only blocked dopamine stimulation of MS activity in D4R-expressing cells but also significantly decreased basal enzyme activity for all three receptor subtypes (Table 1). In contrast, addition of dopamine or L-745870 directly to the MS assay had no effect (data not shown).

Together, these results indicate that basal MS activity is suppressed by D4R expression, but dopamine treatment of D4R-expressing cells allows recovery of a portion of HCY-directed MS activity. Opposite effects of dopamine and L-745870 pretreatment suggests that the suppressive influence of D4R expression on MS activity depends on receptor conformation.

### Methionine cycle metabolites

Since D4R-mediated PLM is dependent on MS and other methylation cycle enzymes, we hypothesized that D4R expression and stimulation might affect levels of methionine cycle metabolites. To test this possibility, we examined metabolite levels in CHO cells expressing D4.2R, D4.4R, and D4.7R, as compared to non-transfected WT CHO cells.

**Fig. 2:**
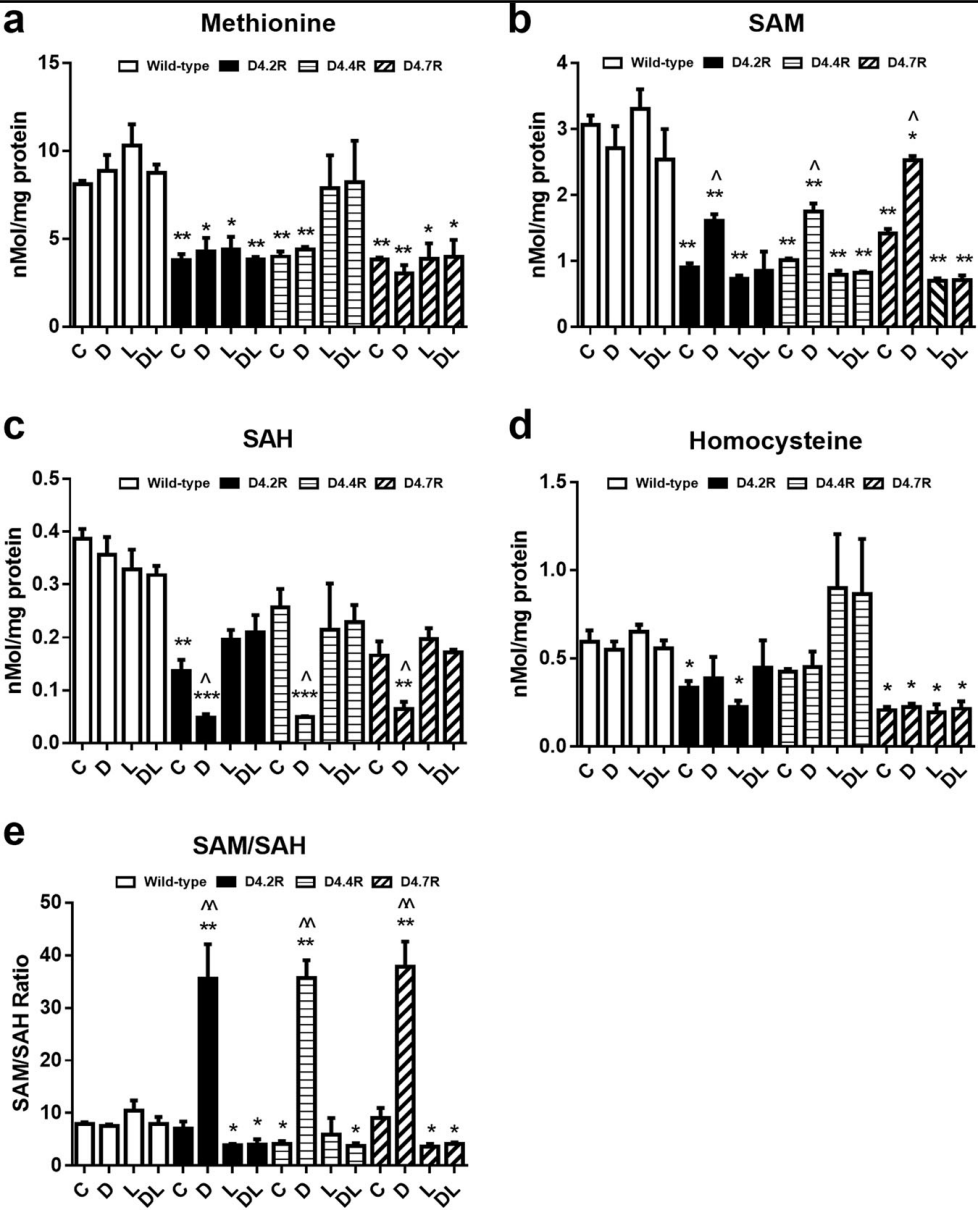
D4R expression and dopamine affect methylation metabolites in CHO cells. Methylation metabolite levels (**a** Methionine, **b** SAM, **c** SAH, **d** Homocysteine, **e** SAM/SAH) were measured in wild-type (WT) CHO cells and CHO cells transfected to express D4.2R, D4.4R, and D4.7R. Cells were treated with dopamine (10 µM; 30 min) (D), or the D4R-selective antagonist L-745870 (100 nM; 60 min) (L) or their combination (DL) (*n* = 3). Data are expressed as mean ± SEM. Asterisks indicate significant differences from matched WT group (*p < 0.05, **p < 0.01). ^ indicates significant difference from untreated control group (C) (^*p* < 0.05, ^^*p* < 0.01)

Consistent with its reduction of MS activity, D4R expression significantly decreased basal methionine levels in CHO cells by 53%, 51%, and 53% in D4.2R-, D4.4R-, and D4.7R-expressing cells, respectively (*p* < 0.005) (Fig. 2a). Dopamine (10 µM; 30 min) had no significant effect on methionine levels, either in D4R-transfected or -non-transfected cells. Treatment with L-745870 (100 nM; 60 min) non-significantly increased methionine in D4.4R-expressing, but not in D4.2R- or D4.7R-expressing cells.

D4R expression significantly decreased basal SAM levels by 71%, 67%, and 54% in D4.2R-, D4.4R-, and D4.7R-expressing cells, respectively, with the decrease being significantly greater for D4.7R vs. D4.2R (*p* < 0.001) or D4.4R (*p* < 0.05) (Fig. 2b). Dopamine treatment increased SAM by 79%, 73%, and 79%, respectively, but had no effect in non-transfected cells. L-745870 blocked the effects of dopamine in all D4R-expressing cells and decreased the basal level of SAM in D4.7R-expressing cells, suggestive of constitutive receptor activity.

D4R expression decreased basal SAH levels by 65%, 34%, and 57% in D4.2R-, D4.4R-, and D4.7R-expressing cells, respectively, whereas dopamine stimulation decreased SAH levels by 65%, 81%, and 41%, respectively (Fig. 2c). Dopamine treatment increased the SAM/SAH ratio by 4.0-, 7.6-, and 3.9-fold in D4.2R-, D4.4R-, and D4.7R-expressing cells, respectively, but did not change the SAM/SAH ratio in non-transfected cells (Fig. 2e). L-745870 decreased the SAM/SAH ratio in D4.7-expressing cells by 60%.

D4R expression significantly decreased basal HCY levels in CHO cells by 44%, and 65% in D4.2R and D4.7R (*p* < 0.05), but the 39% decrease in D4.4R-expressing cells was not significant (*p* = 0.142) (Fig. 2d). Dopamine had no significant effect on HCY levels either in D4R-transfected or -non-transfected cells.

### GSH synthesis pathway metabolites

**Fig. 3:**
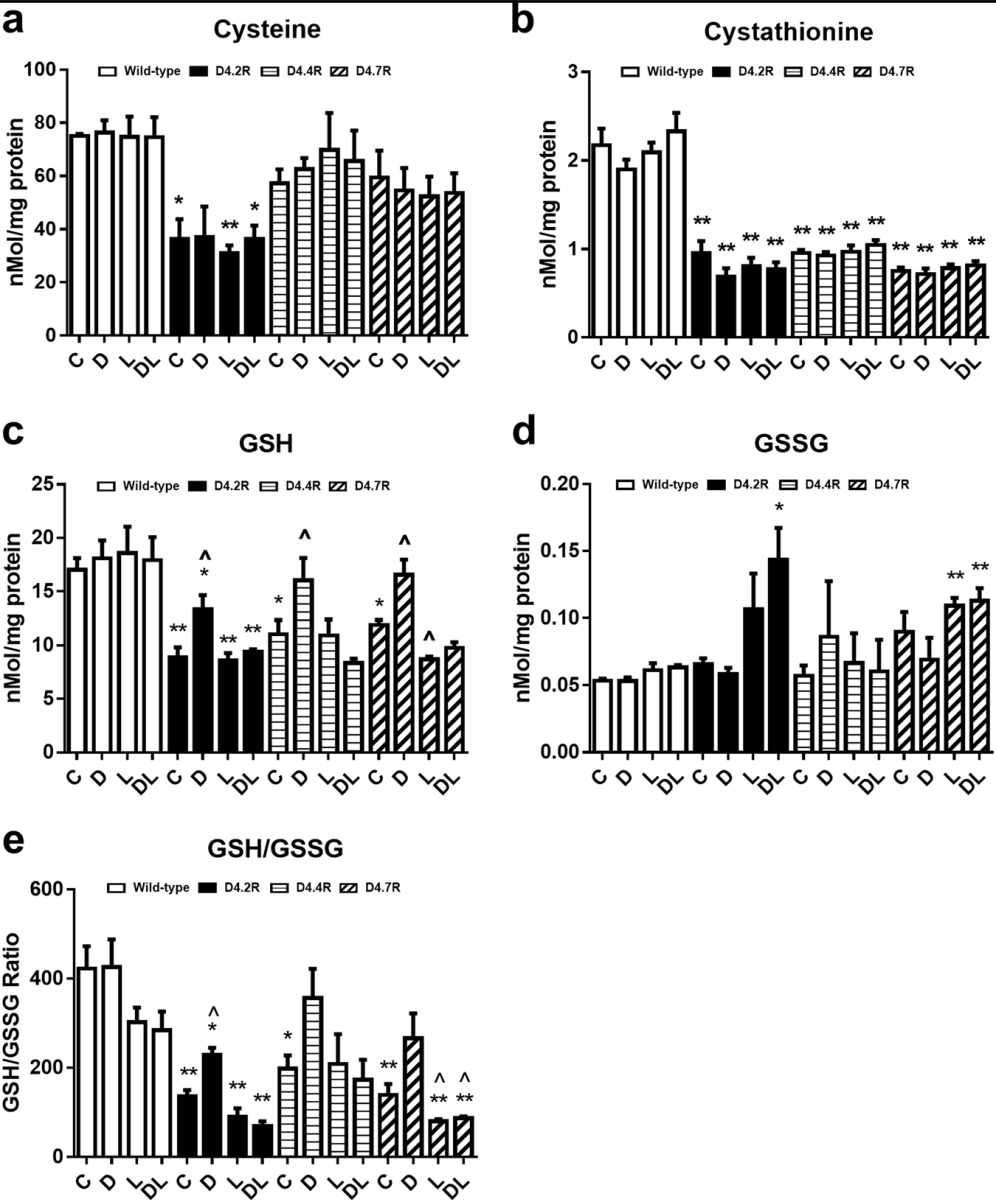
D4R expression and dopamine alter redox-related thiol metabolites in CHO cells. Thiol metabolite levels (**a** Cysteine, **b** Cystathionine, **c** GSH, **d** GSSG, **e** GSH/GSSG) were measured in wild-type (WT) CHO cells and CHO cells transfected to express D4.2R, D4.4R, and D4.7R. Cells were treated with dopamine (10 µM; 30 min) (D), or the D4R-selective antagonist L-745870 (100 nM; 60 min) (L) or their combination (DL) (*n* = 3). Data are expressed as mean ± SEM. Asterisks indicate significant differences from matched WT group (*p < 0.05, **p < 0.01). ^ indicates significant difference from untreated control group (C) (^*p* < 0.05)

Since transsulfuration of HCY to cysteine links the methionine cycle to GSH synthesis, we next examined the effect of D4R expression on metabolites in this redox-related pathway. D4.2R expression significantly decreased basal cysteine levels by 54%, whereas decreases of 24 and 21% for D4.4R and D4.7R, respectively, were not significant (Fig. 3a). The addition of dopamine did not affect cysteine levels in either WT or D4R-expressing cells.

Cystathionine is a key transsulfuration intermediate linking HCy to cysteine. D4R expression significantly decreased cystathionine levels by 56%, 56%, and 65% for D4.2R, D4.4R, and D4.7R, respectively, and its level was unaffected by dopamine or L-745870 (Fig. 3b).

D4R expression significantly decreased basal GSH by 48%, 35%, and 30% in D4.2R-, D4.4R-, and D4.7R-expressing cells, respectively (Fig. 3c). Dopamine treatment increased intracellular GSH by 50%, 45%, and 39% in D4.2R-, D4.4R-, and D4.7R-expressing cells, respectively, but did not affect GSH levels in non-transfected cells. L-745870 blocked the effects of dopamine in all D4R-expressing cells and decreased GSH significantly below basal level in D4.7R-expressing cells. D4R expression did not alter GSSG levels and dopamine treatment had little or no effect (Fig. 3d). Dopamine treatment increased the GSH/GSSG ratio by 69%, 79%, and 90% in D4.2R-, D4.4R-, and D4.7R-expressing cells, respectively, but only D4.4R reached significance (*p* < 0.05) (Fig. 3e). Dopamine did not affect the GSH/GSSG ratio in non-transfected cells.

### Dopamine-stimulated changes in DNA methylation

To investigate whether changes in the observed changes in SAM/SAH ratio translate into downstream changes in methylation, we measured global DNA methylation in D4R-transfected and -non-transfected CHO cells, with or without dopamine treatment. As indicated in Fig. 5, D4.2R, D4.4R, and D4.7R expression decreased basal global DNA methylation by 39%, 30%, and 23%, respectively. Consistent with the increases in SAM/SAH noted above, treatment with dopamine (10 µM; 8 h) increased global DNA methylation level by 137%, 99%, and 102% for D4.2R, D4.4R, and D4.7R variants, respectively. No significant differences were observed among different repeat variants in the extent of increased DNA methylation. The dopamine-induced increase in DNA methylation was blocked by L-745870, and dopamine had no effect on DNA methylation in non-transfected CHO cells. Thus, D4R expression decreases basal DNA methylation and confers DA responsiveness, consistent with its effect on MS activity and SAM/SAH.

## Discussion

Following its initial cloning by Van Tol et al.^22^ in 1991, the D4R received considerable attention for a possible role in schizophrenia,^23–25^ attention-deficit hyperactivity disorder (ADHD),^26–30^ novelty-seeking personality trait,^31–33^ drug abuse liability^34–36^, and longevity.^37,38^ In 1998, we first described the unique ability of the D4R to carry out folate-dependent methylation of membrane phospholipids, utilizing a conformationally active methionine residue at the inner membrane surface.^1^ D4R-mediated PLM requires some of the same enzymes as the classical methionine cycle (Fig. 1a), raising the possibility that it might exert an influence over methionine cycle and interrelated GSH pathway metabolites. Indeed, our current results demonstrate a powerful suppressive influence of D4R expression on MS activity, accompanied by emergence of the ability of dopamine to augment MS activity, and increasing antioxidant and methylation status, including increased DNA methylation. Thus, D4R expression significantly expands the signaling repertoire of dopamine into metabolic and epigenetic domains.

Initial studies indicated that dopamine activates PLM via a conformational movement of transmembrane helix #6 that exposes the active methionine residue (#313 in the D4.4R)^1^, and more recent x-ray crystallography studies subsequently confirmed this large agonist-induced rotational movement for the equivalent leucine residue in β-2 adrenergic receptors.^39^ Since it is the only G protein-coupled receptor (GPCR) with a methionine at this conformationally unique locus, dopamine-stimulated PLM activity appears to be a unique D4R signaling activity. By increasing the bulk and charge density of phospholipid headgroups, PLM alters plasma membrane biophysical properties, which can modulate the activity of membrane proteins sharing the D4R microenvironment.^16,40,41^ The molecular basis for lower PLM activity of the D4.7R may reflect its ability to bind a higher number of membrane proteins through SH3 domain interactions with its expanded repeats. Thus, a higher number of membrane proteins attached to the D4.7R could restrict its access to substrate phospholipids for methylation (Fig. 5a).

The recent demonstration of its binding to PSD-95^16^ indicates that the D4R is well positioned for PLM to affect nearby proteins, as illustrated for glutamate receptors, potassium channels, and Ca^2+^-ATPase in Fig. 5b. In 2003, we first proposed that these actions might underlie attention^42^ and a number of studies have confirmed the ability of D4R activation to promote γ-frequency oscillations in parvalbumin-expressing GABAergic neural networks in association with attention.^43–46^ However, individuals with the D4.7R are at ~4-fold higher risk of ADHD^27–31^ and D4.7R activation induces weaker suppression of GABAergic inhibitory network activity, as compared to D4.4R activation.^16^ Our finding that maximal PLM activity of the D4.7R is lower than D4.2R or D4.4R activity (Fig. 1c) provides a possible explanation for D4.7R-associated ADHD risk.

**Fig. 4:**
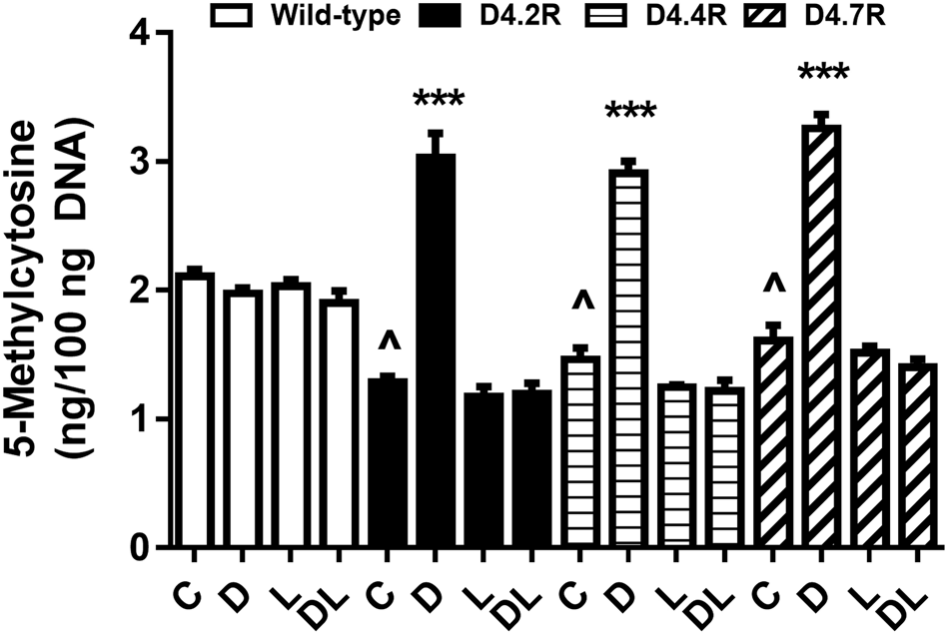
Effects of D4R expression and dopamine on global DNA methylation in D4R-transfected CHO cells. Global DNA methylation was measured in wild-type (WT) CHO cells or CHO cells transfected with D4.2R, D4.4R, or D4.7R. Cells were untreated (C) or treated for 8 h with either dopamine (D; 10 µM) or the D4-selective antagonist L-745870 (L; 100 nM) or both (DL) (*n* = 6). Asterisks indicate significant differences from matched WT group (***p < 0.01). ^ indicates significant difference from untreated control group (C) (^*p* < 0.05)

**Fig. 5:**
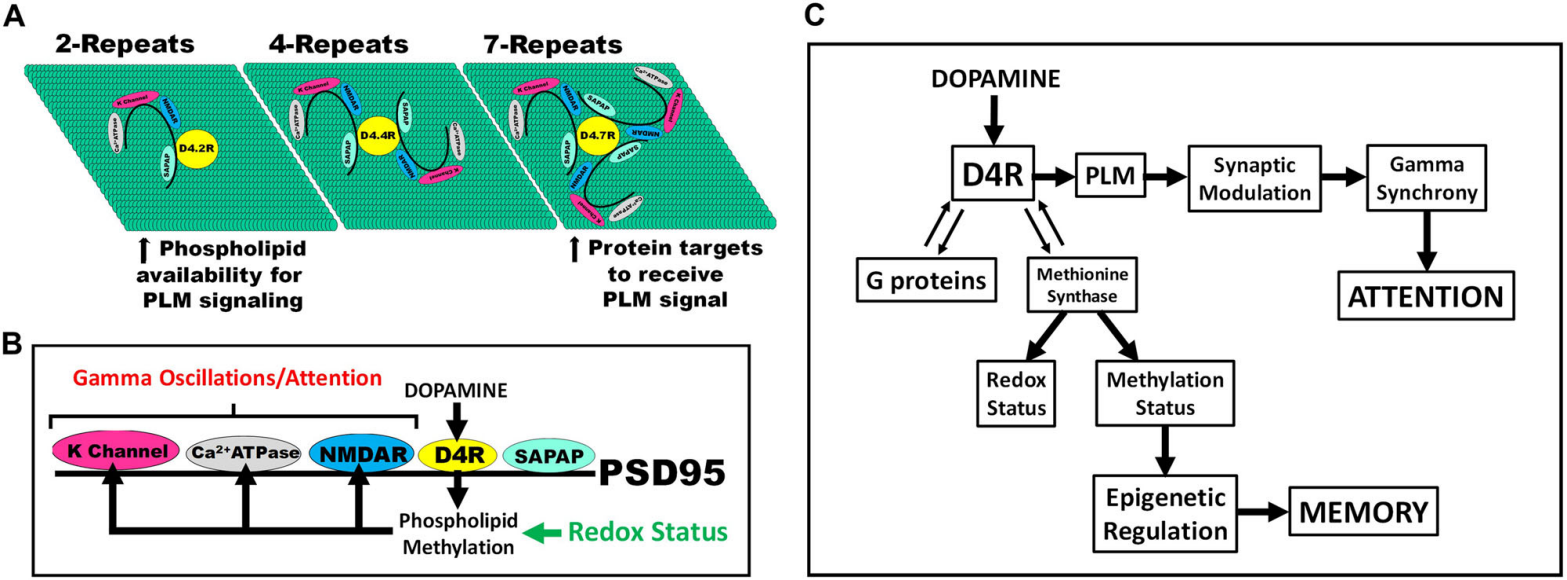
Multiple signals generated by D4R-mediated PLM. **a** Increased interaction with other PSD-bound membrane proteins can restrict access of the D4.7R to substrate phospholipids, thereby restricting the efficiency of D4.7R-mediated PLM. **b** Within its membrane microenvironment, D4R-mediated PLM can modulate activities of other PSD-bound proteins, promoting γ-frequency oscillations associated with attention. Vulnerability of the vitamin B12 cofactor in methionine synthase to oxidation makes D4R-mediated PLM highly sensitive to redox status. **c** D4R-mediated PLM can exert metabolic effects on redox and methylation status, which are thus coordinated with attention. Methylation-dependent epigenetic regulation of gene expression can link memory formation to attended information

D4R expression decreased basal methionine and SAM levels by more than 50%, and dopamine exposure increased the SAM/SAH ratio by 3.9- to 7.6-fold (Fig. 2e). D4R expression therefore allows dopamine to exert profound effects on methylation status, reflected in part as increased DNA methylation (Fig. 4), while other methylation reactions can also be expected to respond to the dopamine-induced increase in SAM/SAH. Thus, D4Rs can exert an extraordinarily broad influence over cellular metabolism, including epigenetic regulation of gene expression, consistent with the recent demonstration that changes in PLM activity significantly alter histone methylation.^47^ As illustrated in Fig. 5c, the metabolic effects of D4R activation provide a link between attention-related γ-frequency synchronization and epigenetic memory formation, representing a potential mechanism for attention-driven learning.

Recognition of the ability of D4R expression and PLM activation to modulate MS activity, GSH levels, the SAM/SAH ratio, and DNA methylation has potential implications for the clinical management of schizophrenia, ADHD, autism, and other neuropsychiatric disorders. It remarkably integrates multiple theories of schizophrenia etiology involving abnormal methylation and single-carbon metabolism,^48–50^ dopamine,^51–53^ and oxidative stress,^54–56^ while D4R modulation of NMDAR activity links these factors to glutamate-based theories. Importantly, oxidative stress and impaired methylation are amenable to metabolic interventions. For example, our previous finding that the level of methylcobalamin (methylB12; the active form of vitamin B12 supporting MS activity) is decreased by ~60% in postmortem frontal cortex of schizophrenia and autism subjects^57^ strongly suggests that normalizing its brain levels may have therapeutic benefit. Several clinical studies have reported modest benefit of methylcobalamin in autism,^58–60^ but none have been conducted in schizophrenia. Adjunctive use of single-carbon folate derivatives, such as folinic acid or l-methylfolate,^61–65^ as well as *N*-acetylcysteine,^66–68^ which augments GSH levels, has been associated with clinical improvement. PE, the substrate for D4R-mediated PLM, is rich in Ω-3 fatty acids, implying that a portion of the therapeutic benefits provided by fish oil supplements may be attributable to normalization of D4R-mediated PLM. While hyperhomocysteinemia is a useful indicator of low systemic MS activity, normal plasma levels do not necessarily exclude a brain deficit.

Our findings are subject to several important limitations. D4R expression and activation in CHO cells might not necessarily reflect the responses occurring in neuronal cells or within the postsynaptic density environment. The metabolic effects we observed might be dependent on the level of D4R expression, which was relatively high in the CHO cell model we employed. While changes in global DNA methylation strongly suggest D4R-mediated epigenetic effects, follow-up studies of gene expression are required to characterize effects on specific genes.

In summary, D4R expression decreases MS-dependent methylation of HCY, affecting levels of key methylation and redox metabolites, while enabling an influence of dopamine over methylation and redox status, including DNA methylation. These previously unreported D4R activities may be important in attention-related memory formation and their dysfunction may contribute to neurocognitive disorders. Optimization of metabolic factors promoting methylation may be a useful adjunctive treatment for these conditions.
